# Is peanut causing food allergy in Cuba? Preliminary assessment of allergic sensitization and IgE specificity profile to peanut allergens in Cuban allergic patients

**DOI:** 10.1186/s40413-017-0156-1

**Published:** 2017-07-11

**Authors:** Mayteé Mateo-Morejón, Alexis Labrada-Rosado, Damaris Torralba-Averoff, Rayza Cruz-Jimenez, Yunia Oliva-Díaz, Mirta Álvarez-Castelló, Alexander Ciria-Martín, Marlene Jiménez-Frandín, Mary Carmen Reyes-Zamora, Raúl Lázaro Castro-Almarales, Beatriz Tamargo-García

**Affiliations:** 1 Allergen Department, Centro Nacional de Biopreprados, Carretera Beltran Km 1 ½, Bejucal, Mayabeque Cuba; 2 Allergology Department, University Hospital “General Calixto García”, Havana, Cuba; 3 Allergology Department, Pediatric Hospital “William Soler, Havana, Cuba; 4 Allergology Department, Pediatric Hospital “Arturo Aballí”, Havana, Cuba; 5 Institute of Pharmacy and Foods, Havana University, Havana, Cuba

**Keywords:** Allergic sensitization, Peanut allergens, Food allergy, IgE specificity

## Abstract

**Background:**

Peanut allergy is increasing at an alarming pace in developed countries. Peanut (*Arachis hypogaea*) is a common food in Cuba. Nevertheless, reported values of sensitization and symptom severity are usually low. As our objective, we carried out an evaluation of allergic sensitivity to perform an assessment of allergic sensitization and IgE specificity profile to peanut allergens in Cuban allergic patients.

**Methods:**

The Skin Prick Test (SPT) was performed for each patient, using two glycerinated allergenic extracts, prepared from raw or roasted peanuts. Overall, 316 food allergic patients (159 adults and 157 children) attending allergy services at four hospitals in Havana were included, as well as 303 adult non- allergic volunteers. The IgE binding profile of 26 selected SPT positive patients was further analyzed by immunoblotting.

**Results:**

The prevalence of sensitization to peanut was 4.6% in general adult population, whereas in adult food-allergic patients it was 18.6%. Prevalence rates were even greater in food allergic children achieving 25.8%. Sensitization frequencies were apparently greater for roasted, as compared to raw peanuts, although the difference was not significant (*p*> 0.05, Mc Nemar's). IgE binding was shown mostly by the 15 and 17 kDa bands, tentatively identified as the major allergens Ara h 2 and Ara h 6. The IgG4 binding profile was similar to IgE, although with more prominence of the bands at 37 and 28 KDa, corresponding to an Ara h 3 fragment and Peanut Agglutinin.

**Discussion:**

The study estimated a relatively high prevalence of peanut sensitization in population. Data reported here suggest that IgE sensitization in Cuban patients is focused mostly on MW bands corresponding to the major allergens Ara h 6 and Ara h 2.

**Conclusions:**

Sensitization to peanut allergen is indeed relatively frequent in Cuba. The IgE profile is congruent to a sensitization pattern by ingestion of roasted peanuts and is directed to well-known major allergens.

## Background

The prevalence of food allergy all over the world is increasing [1–3]. It is estimated that, in children, it has increased by more than 100% in both developed and developing countries in recent years. Peanuts classify among the most allergenic foods. However, there can be differences between the frequency and severity of allergic reactions to food in different geographical regions, which could be related to food culture, climate, environment or genetic factors [4]. Additionally, cross-reactive sensitization to some pollen and aeroallergens also could be associated to the rise of sensitization to foods, and particularly to peanut, in certain regions [5]. The prevalence of peanut allergy has increased in recent years and varies between 0.6 and 1.8% in several developed countries, up to 3% in children in Australia [1, 2, 6–8]. Particularly, allergic reactions to peanut can occur early in life and range from mild oral allergy syndrome to severe anaphylactic reactions.

In Cuba, peanut is a common food, which is eaten usually as roasted seeds. Although peanut is not normally recommended as an early food in infants, it is common practice to be incorporated in the diet starting from 12 months of age. The prevalence of sensitization to food allergens in Cuba has rarely been evaluated systematically. The limited availability of standardized food extracts for diagnostic use in clinical services is a handicap in that regard. Moreover, there are no published data on the profile of IgE recognition to different allergenic peanut proteins. Therefore, the aim of this work was to characterize the allergic immune response to peanut proteins in Cuban patients regarding frequency and intensity of sensitization and IgE binding profile towards components of allergen extracts.

## Methods

### Allergenic extracts

Two peanut allergen extracts were prepared: one from roasted seeds and the other from raw grains. The source material was 200 g of seeds in each case. Roasting was performed at 100 °C for 2 h. Beans were ground and defatted with diethyl ether in a Soxhlet apparatus. Extraction was performed in phosphate buffered saline solution (PBS: NaCl 0.145 mol/L; Na_2_HPO_4_ 0.0085 mol/L; KH_2_PO_4_ 0.0044 mol/L) pH 7.4 at a ratio of 1:20 w/v with mild stirring during 6 h. Next, a process of diafiltration-concentration with Amicon hollow fiber cartridges was performed using a cut-off of 10 kDa. The extract was concentrated and then, diafiltered with PBS. Finally, it was concentrated again (2 fold) to a final volume of 1 L. This volume was sterilized by filtration and subsequently adjusted to a protein concentration of 1 mg/mL by adding PBS. One part was lyophilized and the other formulated adding sterile Glycerol 1:2 w/v. This glycerinated extract was used for Skin Prick Test. The extracts were prepared under aseptic conditions, and stored at 4 °C. Characterization and quality control included SDS-PAGE (Laemmli) [9], protein content assay (Lowry) [10], and sterility test, as well as glycerin content by titration with Sodium Periodate solution [11].

### Patient selection

The study was conducted following the ethical principles contained in the Declaration of Helsinki [12]. Subjects were asked for their written informed consent to participate in the study and, in the case of children, parental consent was requested. The study was approved by the ethics committee of each of the hospitals in Havana (University Hospital “General Calixto García”, University Pediatric Hospitals “William Soler” and “Angel Arturo Aballí” and the “Hermanos Ameijeiras” Clinical Hospital).

Selection of patients was based on clinical history of allergic symptoms upon consumption of the food, as reported by the patient himself. Patients were selected among those who attended the allergy service of four hospitals in Havana (University Hospital “General Calixto García”, University Pediatric Hospitals “William Soler” and “Angel Arturo Aballí” and the “Hermanos Ameijeiras” Clinical Hospital). Overall, 316 patients (159 adults, above 16 years old and 157 children between 2 and 16 years old) were recruited. In addition to allergic patients, 303 adult volunteers who had no signs suggestive of food allergy were selected as the control group.

### Skin Prick Test (SPT)

SPT was practiced to all the recruited subjects. Both, glycerinated extracts of roasted and raw peanut were used. To carry out the SPT, subjects were instructed to suspend any medication plan that could influence the results. The test was conducted between 8:00 am and 10:00 am, always, to avoid circadian variations. Stainless steel lancets with tips of 1 mm (ALK, Denmark) were used according to international standards. Histamine HCl 10 mg/mL was used as a positive control, and as a negative control PBS/glycerol. In order to record the test results, a line was drawn around the wheal and transferred to a transparent adhesive tape, which was taped on to a data recording book. The largest and the orthogonal diameters were measured on the wheal drawing, and the mean diameter was calculated. Furthermore, the mean diameter (d) between the two arms was calculated.

The test was considered valid if the difference between both arms wheals was less than 2 mm for wheals between 3 and 6 mm, or less than 3 mm for larger wheals; besides being positive for Histamine HCl (positive control) and negative for the diluent solution (negative control). The test was considered positive for d ≥ 3 mm.

### SDS-PAGE and IgE/IgG4 Immunoblotting

SDS-PAGE followed by immunoblotting was performed for testing IgE and IgG4 serum reactivity to different molecular weight components of the extract. Only roasted peanut extract was used in this method. For this purposed 10 mL of blood was extracted of several SPT positive selected patients. First, SDS-PAGE was performed according to Laemmli. Briefly, extract samples were run in reducing buffer (with ß-mercaptoethanol), after heating for 5 min at 100 °C. A 12.5% acrylamide gel was used. In parallel to immunoblotting, staining of gels with Coomassie blue R-250 was used for visualizing protein bands. For identification of IgE-binding proteins, the gel was subjected to electrophoretic transfer to a nitrocellulose membrane 0.45 μm during 2 h using semi-dry transfer equipment (Pharmacia). Then, blocking of non-specific sites of the membrane was performed with PBS Tween 0.5% BSA 1% for 1 h at room temperature. Afterwards, the membrane was incubated with patient’s sera diluted 1:2 overnight, with mild stirring at 4 °C. After washing with PBS-Tween 0.1%, a monoclonal anti human IgE conjugated to Alkaline Phosphatase (Sigma, USA) was applied at a 1:1000 dilution and incubated for 1 h at RT with slow stirring. Finally, the colorimetric substrate 5-bromo-4-chloro-3-indolyl phosphate BCIP/NBT (Sigma, USA) was added. To determine IgG4 binding proteins, the same procedure was used, adding a monoclonal anti-human IgG4 conjugated with Biotin (Sigma) at a dilution of 1:1000 and incubated for 1 h at RT. Afterwards, Streptavidine Peroxidasa (Sigma) was added at a 1: 10 000 dilution, and incubated at RT for 30 min, and finally, Diaminobenzidine (Sigma) was used as the colorimetric substrate.

### Statistics

Data processing was performed using statistics tools of MICROSOFT EXCEL, version 7.0 and the statistical add-inXLSTAT version 1.02, as well as the statistical packages STATISTICA version 4.0 and Graphpad Prism version 4.0. For the analysis of the SPT data (wheal diameter) parametric methods (Pearson correlation coefficient and ANOVA) were used. For categorical variables (Frequency of sensitization, allergic manifestations in SPT positive patients), the non-parametric McNemar’s test was used.

## Results

**Fig. 1:**
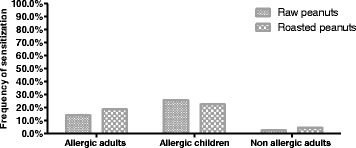
Frequency of sensitization to peanut (positive SPT to raw or roasted allergen extracts) by groups

Two variants of peanut extract were used for SPT, one prepared from raw peanuts and the other from roasted peanuts. Patient’s reactivity was analyzed in three groups: allergic adults, allergic children and non-allergic controls (only adults). No significant differences in terms of sensitization (i.e. frequency of positive SPT) was detected between the two extract variants for any of the analyzed groups (McNemar’s *p* = 0.511) (Fig. 1). The group of allergic children was the only approaching to a significant value (*p* = 0.063) suggesting slightly greater sensitization values to the roasted variant (25.8%) as compared to the raw one (22.6%).

**Fig. 2:**
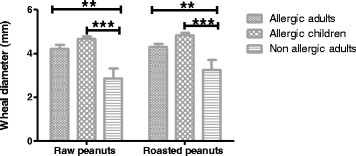
Geometric mean of the SPT wheal diameterin individuals with positive test result to raw or roasted peanut extracts. Sign. values are indicated between allergic adults and the non-allergic group, particularly *** (*p* = <0.0001) and between allergic children and the control group ** (*p* = <0.001)

Allergic adults showed lower frequencies of sensitization: 18.6 and 14.2%, to roasted and raw variants, respectively, as compared to allergic children: 25.8 and 22.6% (Fig. 1), suggesting that the prevalence of sensitization decreases with age. However, in terms of intensity of sensitization as measured by the size of the skin response, in SPT positive patients, no significant differences between allergic adults and children were noted (two way ANOVA *p* > 0.05); whereas, as expected, highly significant values were obtained between allergic and non-allergic adults (*p* <0.001), and between allergic children and non-allergic adults (*p* <0.0001) (Fig. 2). In the later control population, the prevalence of allergic sensitization was 2.6% to raw and 4.6% to roasted peanuts.

**Fig. 3:**
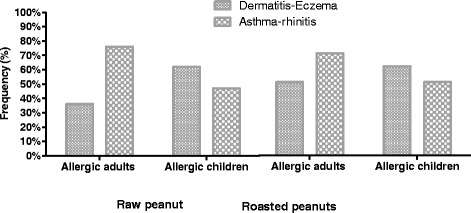
Frequency of clinical allergic manifestations by group in SPT positive patients to roasted and raw peanut extracts

As for allergic reactions within reported reactions to peanut, skin manifestations (dermatitis and/or eczema) were the most frequent in children, whereas respiratory symptoms predominated in adults, with a similar behavior to both extract variants. These manifestations were self-reported by the patient, so no direct relationship could be established between clinical symptoms and ingestion or exposure to peanut containing foods (Fig. 3).

**Fig. 4:**
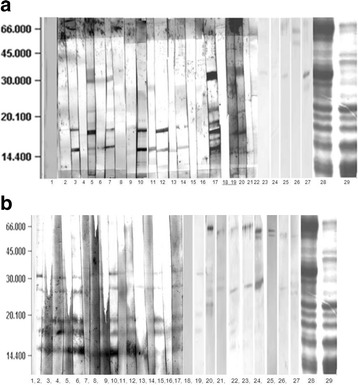
Western Blotting of roasted peanut extracts: **a**: IgE binding profile, **b**: IgG4 binding profile. MWM: Molecular Weight markers. Lanes 1: HSA 0.5% as negative control, Lanes 2 to 19, 21, 23 to 25 and 27, allergic adults, Lanes 20, 22 and 26 allergic children: Lanes 28 and 29: SDS-PAGE Coomassie stained gels of raw and roasted peanut extracts, respectively

Prior to immunoblotting, Coomassie stained SDS-PAGE gels showed a wide spectrum of protein bands between 60–66, 37 and 17–21 kDa (Fig. 4 a and b). Both variants showed a similar pattern of bands in the low molecular weight region (14–37 kDa), whereas the roasted extract showed a diminished intensity of the bands at higher molecular weights between 37 and 66 KDa, which could be caused by protein denaturation.

The immunoreactivity profile to different protein components of peanut extracts was assessed by immunoblotting using sera of 26 SPT-positive patients (3 children and 23 adults). In spite of the differences in the SDS-PAGE Coomassie stained gels, no differences were noted by immunoblotting between roasted and raw peanut extracts. Figure 4 shows results of the IgE and IgG4 immunoblotting using the roasted variant.

IgE antibodies recognized at least nine different components at the molecular weights of 10, 15, 16, 17, 18, 22, 37, 60 and 64 kDa (Fig. 4, Table 1). Remarkably, one patient (number 17) recognized a peak value of seven bands. This patient suffered from a severe generalized eczema.

**Table 1:** IgE and IgG4 binding frequency by SDS-PAGE Western Blotting among selected allergic patients

Band MW	IgE binding frequency, %	IgG4 binding frequency, %	Putative ID (www.allergen.org)
10	3.8	0	Ara h 9
15	57.7	46	Ara h 5, **Ara h 6**, Ara h 7, Ara h 11
16	3.8	0	Ara h 7, Ara h 8, Ara h 10
17	53.8	34.6	**Ara h 2**, Ara h 7, Ara h 8, Ara h15
18	3.8	38.4	Ara h 7, Ara h 14, Ara h 15
28	15.3	53.8	Peanut agglutinin (PNA), lectin
37	30.7	65.3 (high intensity)	**Ara h 3.02** (Ara h 3 fragment)
60	7.7	15.3	Ara h 1, **Ara h 3**
64	3.8	19.5	Ara h 1

Higher IgE binding frequency showed the bands at 15 (57.7%), 17 (53.8%) and 37 kDa (30.7%). Since the insufficient resolution and accuracy of the SDS-PAGE separation method is was not possible to unambiguously identify the bands corresponding to the reported allergenic molecules. Table 1 shows the IgE and IgG4 binding frequencies as assessed by SDS-PAGE Western Blotting among selected allergic patients, to roasted penut proteins. Ara h 2, which is regarded the most clinically relevant peanut allergen could be possibly contained in the 17 kDa band, whereas the major allergen Ara h 6 could be located in the 15 kDa band [13].

The IgG4 binding pattern was very similar, although the number of bands was slightly lower: seven, at the following MWs: 15, 17, 18, 22, 28, 37, 60 and 64 kDa (Fig. 4, Table 1). The bands more recognized were found at 15 (46%), 17 (34.6%), 18 (38.4%), 28 (53.8%) and 37 kDa (65.3%). The later, presumably an Ara h 3 fragment, showed also the highest intensity. Striking differences as compared to the IgE binding frequency were found for the bands at 18 kDa (38.4% for IgG4 vs 3.8% for IgE) and, to a lesser extent, for the 28 kDa band (53.8 vs. 15.3%).

## Discussion

In spite of the fact that allergy to peanut is not perceived in Cuba as a frequent or serious health problem, the study estimated a relatively high prevalence of peanut sensitization in the general adult population (4.8%; 95% CI: 2.3–7.2%). This value is higher than that reported internationally in several developed countries, including US, UK, Sweden and Germany (between 0.6 and 1.8%) [1, 2, 6–8]. Values of sensitization frequency to peanut among patients with symptoms suggestive of food allergy were also high, and were higher in children (25.8%) than in adults (18.6%), a fact that reasonably agrees with the well-known process of tolerance induction towards food allergens. However, in spite of these high sensitization values, the severity of allergic manifestations does not seem alarming in our country, according to the generalized opinion of allergy practitioners. There are almost no case reports of anaphylactic shocks to peanut in contrast to other countries [13]. This contradiction is intriguing and could possibly be related to the prevailing forms of exposure to this food, and specifically, the preferential consumption of roasted seeds and the relative low exposure to other peanut containing foodstuff, common in industrialized countries, like peanut butter.

Elucidating the full spectrum of IgE reactivity can provide clues on the sensitization process in a given population. Data reported here suggest that IgE sensitization in Cuban patients is focused mostly on MW bands corresponding to the major allergens Ara h 6 (15 KDa) and Ara h 2 (17 kDa). Ara h 2, a peanut trypsin inhibitor, belong to the so-called “Class I” peanut allergens [14], which are stable against denaturation via digestion and thermal processing, therefore are able to induce sensitization via the gastrointestinal tract and to elicit systemic clinical symptoms. Other allergens that belong to Class I are Ara h 1 and Ara h 3 [8, 12, 13].

On the other hand, sensitization to Class II peanut allergens would be caused, preferentially, by cross-reactivity toward respiratory allergens such as pollen. A hallmark of this sensitization pattern is the allergen Ara h 8 (16 KDa), a protein cross-reactive with the major birch pollen allergen Bet v 1. Since pollinosis in tropical countries is not as important as in temperate areas, it would not be expected to find a high reactivity to these Class II allergens. However, the highly reactive 15 KDa band, as described here, is likely to contain Ara h 5, Ara h 6 and Ara h 7, allergens that have been also classified by some authors as Class II [11]. Like Ara h 2, Ara h 6 is resistant to digestion and stable against heating, especially in baking process [15], and it is reported as the most clinically important peanut allergen by several authors, in studies performed in temperate countries, in which birch pollinosis is frequent [16] Both, Ara h 6 and Ara h 7 belong to the Prolamin superfamily, very similar to Ara h 2. Moreover, Ara h 6 shares 53% homology to Ara h 5, which is a member of the Profilin family, a well-known pan-allergen, responsible for cross-reactivity between food and pollen [12, 17, 18]. Overall, the frequencies of sensitization reported here for the bands at 15 and 17 kDa, did no differ markedly to what has been reported for populations in temperate countries (Europe, North America, Japan) to the allergens Ara h 2 and Ara h 6, which have shown IgE binding frequencies ranging roughly from 50 to 100% in food allergic patients exposed to peanut [19]. Although, in this case, the high frequency of sensitization to Ara h 6 cannot be attributed to pollen sensitization, and other possible causes, such as intrinsic biologic activity and resistance to digestion, should be taken into account in order to explain this finding.

The fact that sensitization in Cuba should arise upon exposure to roasted peanut by ingestion is also congruent with a sensitization profile towards heat-stable allergens, such as Ara h 2 and Ara h 6. In fact, a study in 2005 [20] showed that thermal processing of this allergen induced modifications in certain epitopes subjected to Maillard glycation reaction which are responsible for increasing allergenicity. Beyond the 15 and 17 KDa bands, 8 patients (30.7%) showed IgE binding to a 37 kDa band, which could correspond to an isoform of the Ara h 3 allergen (termed Ara h 3.02). This allergen, previously known as Ara h 4, is in fact a fragment or subunit of Ara h 3 (60 kDa), a seed storage protein (vicilin) that is cleaved under SDS-PAGE reducing conditions [21]. The resistance against digestion has been suggested as a cause of Ara h 3/4 allergenicity [3].

Other low molecular allergens such as Oleosins (Ara h 10, Ara h 11, Ara 14 and Ara h 15) are not likely to contribute significantly to the IgE activity of the extracts, since the aqueous extraction method used here favors the content of hydrophilic proteins and not the lipophilic ones [13]. Similarly, the 10 KDa cutoff limit, applied during extract ultra filtration, does not seem likely to affect its allergenic activity in this study, since only the minor allergens Ara h 9, Ara h 12, Ara h 13, Ara h 16 and Ara h 17 have known MW lower than 10 KDa, and among them, only Ara h 9 (9.1 KDa), has shown some relevance regarding allergic sensitization, specifically in Mediterranean countries [21]. However, it is also a Class II allergen, owing its sensitization to previous exposure to peach or hazelnut in the diet. In fact, according to published data, Pru p 3 has shown a strong capacity to inhibit IgE-binding to Ara h 9 and Cor a 8, while Ara h 9 and Cor a 8 are unable to inhibit IgE-binding to Pru p 3, which suggests that, indeed, Pru p 3 acts as the primary sensitizer in these populations [22]. Both, peach and hazelnut are absent in the common Cuban diet.

As opposed to IgE, allergen specific IgG4 antibodies are recognized as a tolerance marker towards food allergy. Thus, the IgE/IgG4 balance could hypothetically have some value as a predictor or allergy severity or disease evolution. However, the IgG4 binding profile to allergen extracts can differ substantially from the IgE binding pattern, and these differences can be associated to clinical symptoms [23]. Overall, in this work, a very similar repertoire of peanut specific IgG4 as compared to IgE recognition could be noticed, although the frequencies and intensities were different. Particularly, the 18 kDa band (Ara h 7) showed a 10-fold increase in binding frequency (from 3.8 to 38.4%); the 28 KDa band (Peanut Agglutinin, PNA) also increased notably its binding frequency (up to 53.8%) and the band at 37 kDa (Ara h 3 fragment) achieved the highest frequency and intensity, surpassing the major allergens Ara h 2 and Ara h 6. It is worth to note that the IgE binding activity of PNA has also been associated to Maillard reactions after heat treatment, although data are contradictory and one author indicated that IgE affinity seems to deteriorate by non-enzymatic browning reactions [20]. Interestingly, it seems that reactivity to the 37 kDa band tends to polarize to either IgE or IgG4 antibody response, since only 2 out of 14 patients showed simultaneous positivity to both antibody classes. More data are needed to assess whether IgG4 response to certain peanut proteins could be used as a marker of increased tolerance or a less severe allergic disease.

## Conclusion

The results indicate that sensitization to peanut allergen is indeed relatively frequent in Cuba both in children and adults, suggesting that this sensitization is linked to exposure mostly to roasted peanut and directed to well-known heat-stable major allergens such as Ara h 2, Ara h 6 and Ara h 3. No clear explanations are derived from this pattern to support the apparent low severity of allergic systemic reactions to peanut in the Cuban population. Skin testing, in conjunction with controlled food challenges, should be considered a useful tool for improving the specific diagnosis of clinical food allergy in Cuba. More studies using molecular diagnostic tools are needed to accurately elucidate the full spectrum of IgE specificity in the Cuban population.

## Abbreviations

Ig: Immunoglobulin
PBS: Phosphate buffered saline
SDS-PAGE: Polyacrylamide gel electrophoresis
SPT: Skin prick test
